# Pediatric severe asthma: a case series report and perspectives on anti-IgE treatment

**DOI:** 10.1186/s12887-018-1019-9

**Published:** 2018-02-21

**Authors:** Virginia Mirra, Silvia Montella, Francesca Santamaria

**Affiliations:** Department of Translational Medical Sciences, Federico II University, Via Sergio Pansini 5, 80131 Naples, Italy

**Keywords:** Severe asthma, Omalizumab, Children, Adolescents, Asthma exacerbations

## Abstract

**Background:**

The primary goal of asthma management is to achieve disease control for reducing the risk of future exacerbations and progressive loss of lung function. Asthma not responding to treatment may result in significant morbidity. In many children with uncontrolled symptoms, the diagnosis of asthma may be wrong or adherence to treatment may be poor. It is then crucial to distinguish these cases from the truly “severe therapy-resistant” asthmatics by a proper filtering process. Herein we report on four cases diagnosed as difficult asthma, detail the workup that resulted in the ultimate diagnosis, and provide the process that led to the prescription of omalizumab.

**Case presentation:**

All children had been initially referred because of asthma not responding to long-term treatment with high-dose inhaled steroids, long-acting β_2_-agonists and leukotriene receptor antagonists. Definitive diagnosis was severe asthma. Three out four patients were treated with omalizumab, which improved asthma control and patients’ quality of life. We reviewed the current literature on the diagnostic approach to the disease and on the comorbidities associated with difficult asthma and presented the perspectives on omalizumab treatment in children and adolescents. Based on the evidence from the literature review, we also proposed an algorithm for the diagnosis of pediatric difficult-to-treat and severe asthma.

**Conclusions:**

The management of asthma is becoming much more patient-specific, as more and more is learned about the biology behind the development and progression of asthma. The addition of omalizumab, the first targeted biological treatment approved for asthma, has led to renewed optimism in the management of children and adolescents with atopic severe asthma.

## Background

Children with poor asthma control have an increased risk of severe exacerbations and progressive loss of lung function, which results in the relevant use of health resources and impaired quality of life (QoL) [1]. Therefore, the primary goal of asthma management at all ages is to achieve disease control [2–4].

According to recent international guidelines, patients with uncontrolled asthma require a prolonged maintenance treatment with high-dose inhaled corticosteroids (ICS) in association with a long-acting β_2_-agonist (LABA) *plus* oral leukotriene receptor antagonist (LTRA) (Table 1) [5].

**Table 1:** Recommended options for initial controller treatment in children and adults according to GINA Guidelines [5]

	Step 1	Step 2	Step 3	Step 4	Step 5
Preferred choice	–	Low dose ICS	Low dose ICS/LABA	Medium/high dose ICS/LABA	Add anti-IgE
Alternative choices	Low dose ICS	LTRA	Medium/high dose ICS	Add tiotropium	Add tiotropium
		Low-dose theophylline	Low dose ICS + LTRA	High dose ICS + LTRA	Add low dose OCS
			Low dose ICS + theophylline	High dose ICS + theophylline	

Theophylline is not recommended for children 6–11 years, while tiotropium is not indicated in patients < 18 years

*ICS* inhaled corticosteroids, *LTRA* leukotriene receptor antagonist, *LABA* long-acting β_2_-agonist, *anti-IgE* anti-immunoglobulin E therapy, *OCS* oral corticosteroids

Nevertheless, in the presence of persistent lack of control, reversible factors such as adherence to treatment or inhalation technique should be first checked for, and diseases that can masquerade as asthma should be promptly excluded. Finally, additional strategies, in particular anti-immunoglobulin E (anti-IgE) treatment (omalizumab), are suggested for patients with moderate or severe allergic asthma that remains uncontrolled in Step 4 [5].

Herein, we reviewed the demographics, clinical presentation and treatment of four patients with uncontrolled severe asthma from our institution in order to explain why we decided to prescribe omalizumab. We also provided a review of the current literature that focuses on recent advances in the diagnosis of pediatric difficult asthma and the associated comorbidities, and summarizes the perspectives on anti-IgE treatment in children and adolescents.

## Case presentations

Table 2 summarizes the clinical characteristics and the triggers/comorbidities of the cases at referral to our Institution. Unfortunately, data on psychological factors, sleep apnea, and hyperventilation syndrome were not available in any case. Clinical, lung function and airway inflammation findings at baseline and after 12 months of follow-up are reported in Table 3. In the description of our cases, we used the terminology recommended by the ERS/ATS guidelines on severe asthma [6].

**Table 2:** Clinical characteristics of described patients with difficult asthma

	Case 1	Case 2	Case 3	Case 4
Age at asthma symptoms onset	3 years	6 years	3 years	4 years
History	Monthly asthma exacerbations/ hospital admissions	Monthly asthma exacerbations/ hospital admissions	Monthly asthma exacerbations/ hospital admissions	Monthly asthma exacerbations/ hospital admissions
	Frequent need of systemic steroids	Frequent need of systemic steroids	Several ICU admissions	Frequent need of systemic steroids
			Frequent need of systemic steroids	
Allergen sensitization	House dust mites, dog dander, *Graminaceae* pollen mix, *Parietaria judaica*	House dust mites	House dust mites, dog and cat dander, *Alternaria alternata*, *Graminaceae* pollen mix, *Artemisia vulgaris*, *Parietaria judaica*, *Olea europaea* pollen, cow milk proteins, egg, peanuts	House dust mites, dog dander, *Graminaceae* pollen mix, *Olea europaea* pollen, tomatoes, beans, shrimps, peas
Age at referral	11 years	10 years	6 years	8 years
Comorbidity	Rhinosinusitis	GER	Absent	Absent
Treatment at referral	Fluticasone (1000 μg/d) + salmeterol + montelukast	Fluticasone (1000 μg/d) + salmeterol + montelukast	Fluticasone (1000 μg/d) + salmeterol + montelukast	Fluticasone (1000 μg/d) + salmeterol + montelukast

*GER* Gastroesophageal reflux, *ICU* Intensive care unit

**Table 3:** Clinical findings at baseline and after 12 months of follow-up in patients with difficult asthma

	Case 1		Case 2		Case 3		Case 4	
	Baseline	T 12 months	Baseline	T 12 months	Baseline	T 12 months	Baseline	T 12 months
FVC (% pred)	109	127	95	97	98	95	103	113
FEV_1_ (% pred)	80	78	74	85	67	94	90	85
Post BD ΔFEV_1_ (%)	3	NA	7	NA	25	NA	12.1	NA
FEV_1_/FVC (%)	64	87	69	73	67	82	70	66
FEF_25–75_ (% pred)	14	61	41	55	25	72	48	46
Post BD ΔFEF_25–75_ (%)	21	NA	8	NA	55	NA	69	NA
FeNO (ppb)	54	21	19	7	36	5	116	NA
QoL score	2.0	6.7	3.9	6.5	6.4	6.8	4.0	5.9
c-ACT Score	17	23	22	25	17	21	12	15
Current treatment	Fluticasone (500 μg/d) + salmeterol + montelukast		Fluticasone (100 μg/d) + salmeterol + omalizumab		Fluticasone (200 μg/d) + montelukast + omalizumab		Fluticasone (1000 μg/d) + salmeterol + montelukast	

*BD* bronchodilator, Δ % predicted changes from the pre-bronchodilator values, *FeNO* fractional exhaled nitric oxide, *Ppb* part per billion, *QoL* Quality of Life defined according to references [14], *c-ACT* Children Asthma Control Test evaluated according to references [79, 80], *NA* Not Available

### Case 1

A full-term male had severe preschool wheezing and, since age 3, recurrent, severe asthma exacerbations with frequent hospital admissions. At age 11, severe asthma was diagnosed. Sensitization to multiple inhalant allergens (i.e., house dust mites, dog dander, *Graminaceae* pollen mix, and *Parietaria judaica*) and high serum IgE levels (1548 KU/l) were found. Body mass index (BMI) was within normal range. Combined treatment with increasing doses of ICS (fluticasone, up to 1000 μg/day) in association with LABA (salmeterol, 100 μg/day) plus LTRA (montelukast, 5 mg/day) has been administered over 2 years. Nevertheless, persistent symptoms and monthly hospital admissions due to asthma exacerbations despite correct inhaler technique and good adherence were reported. Parents refused to perform any test to exclude gastroesophageal reflux (GER) as comorbidity [6]. However, an *ex-juvantibus* 2-month-course with omeprazole was added to asthma treatment [7], but poor control persisted. Anterior rhinoscopy revealed rhinosinusitis that was treated with nasal steroids for six months [8], but asthma symptoms were unmodified. Treatment with omalizumab was added at age 12. Reduced hospital admissions for asthma exacerbations, no further need for systemic steroids, and improved QoL score (from 2.0 up to 6.7 out of a maximum of 7 points) were documented over the following months. Unfortunately, after one year of treatment, adherence to omalizumab decreased because of family complaints, and eventually parents withdrew their informed consent and discontinued omalizumab. Currently, by age 17, treatment includes inhaled salmeterol/fluticasone (100 μg/500 μg∙day^-1^, respectively) *plus* oral montelukast (10 mg/day). Satisfactory symptom control is reported, with no asthma exacerbations.

### Case 2

A full-term male, who had a recurrent severe preschool wheezing, at 6 years of age developed exercise-induced asthma. At age 10, severe asthma was diagnosed. High serum IgE levels (1300 KU/l) and skin prick tests positive to house dust mites were found. Despite a 3-year treatment with progressively increasing doses of inhaled fluticasone (up to 1000 μg/day) combined with salmeterol (100 μg/day) and oral montelukast (5 mg/day), monthly hospital admissions with systemic steroids use were reported. At age 13, a 24-h esophageal impedance/pH study demonstrated the presence of acid and non-acid GER [7]. Esomeprazole was added to asthma medications, but with an incomplete clinical benefit for respiratory symptoms. Esomeprazole was withdrawn after 3 months, and parents refused to re-test for GER. As respiratory symptoms persisted uncontrolled despite treatment, severe asthma was definitively diagnosed [6]. BMI was within the normal range and anterior rhinoscopy excluded rhinosinusitis. Inhaler technique and adherence were good; thus we considered the anti-IgE treatment option [9]. Subcutaneous omalizumab was started, with fast improvement of both symptoms and QoL score (from 3.9 up to 6.5). Seventeen months later, the dose of ICS had been gradually tapered and oral montelukast definitely discontinued. Currently, at age 14, treatment includes the combined administration of bimonthly subcutaneous omalizumab and of daily inhaled salmeterol/fluticasone (50 μg/100 μg∙day^-^^1^, respectively). Asthma control is satisfactory and no side effects are reported. Omalizumab has been continuously administered for 2.6 years and is still ongoing.

### Case 3

A full-term male had severe preschool wheezing and, since age 3, recurrent, severe asthma exacerbations with acute respiratory failure that frequently required intensive care unit (ICU) admission. At age 6, sensitization to multiple perennial inhalant (i.e., house dust mites, dog and cat danders, *Alternaria alternata*, *Graminaceae* pollen mix, *Artemisia vulgaris*, *Parietaria judaica*, and *Olea europaea* pollen) and food allergens (i.e., egg, milk, and peanut) was diagnosed. Serum IgE levels were 2219 KU/l. Weight and height were appropriate for age and sex. The patient has been treated over 3 years with a combined scheme of high-dose inhaled fluticasone (up to 1000 μg/day) *plus* salmeterol (100 μg/day) and oral montelukast (5 mg/day), with correct inhaler technique and good adherence. Despite this, monthly hospital admissions with systemic steroids use were recorded. Rhinosinusitis and GER were excluded on the basis of appropriate testing; thus treatment with omalizumab was started when the patient was 9 years old. At age 11, adherence to treatment is satisfactory, with no side effects. More importantly, reduced hospital admissions for asthma exacerbations, no further need for systemic steroids, and improved QoL score (from 6.4 to 6.8) were reported. Finally, progressive step-down of anti-asthma treatment was started, and at present (by 11.5 years) inhaled fluticasone (200 μg/day) *plus* bimonthly subcutaneous omalizumab provide good control of symptoms. Omalizumab has been continuously administered for 2.6 years and is still ongoing.

### Case 4

A full-term male had severe preschool wheezing and, since age 4, recurrent, severe asthma exacerbations with frequent hospital admissions. At age 8, multiple perennial inhalants and food sensitization (i.e., house dust mites, dog dander, *Graminaceae* pollen mix, *Olea europaea* pollen, tomatoes, beans, shrimps, and peas) and high serum IgE levels (1166 KU/l) were found. The patient has been treated over 5 years with inhaled fluticasone (up to 1000 μg/day) in association with salmeterol (100 μg/day) and oral montelukast (5 mg/day). Despite this, monthly hospital admissions with systemic steroids need were recorded. After checking the inhaler technique and adherence to treatment, comorbidities including obesity, rhinosinusitis and GER were excluded. Omalizumab was proposed, but parents refused it. By 13.6 years, despite a treatment including the association of inhaled salmeterol/fluticasone (100 μg/1000 μg∙day^− 1^, respectively) *plus* oral montelukast (10 mg/day), monthly exacerbations requiring systemic steroids are reported.

## Discussion and conclusions

Most children and adolescents with asthma respond well to inhaled short-acting beta_2_-agonists (SABA) on demand if symptoms are intermittent, or to low dose controller drugs *plus* as-needed SABA if the risk of exacerbations increases [1]. Nevertheless, a proportion of patients is referred to specialists because this strategy is not working and asthma is persistently uncontrolled [4]. For these children, assessment is primarily aimed at investigating the reasons for poor control. Indeed, when the child is initially referred, before the label of “severe, therapy-resistant asthma” (i.e., not responding to treatment even when factors as exposure to allergens and tobacco smoke have been considered) is assigned, three main categories need to be identified: 1) “not asthma at all”, in which response to treatment is suboptimal because the diagnosis is wrong; 2) “asthma *plus*”, when asthma is mild but exacerbated by one or more comorbidities; and 3) “difficult-to-treat asthma”, when asthma is uncontrolled because of potentially reversible factors [10].

The reported cases highlight some aspects of the disease process that may expand the diagnosis and improve patients’ care. At our institution, the severe asthma program includes a multidisciplinary approach with consultations by gastroenterologists as well as ear, nose and throat experts. Recently, sleep medicine experts joined this multidisciplinary team; thus, unfortunately, sleep-disordered breathing (SDB) could not be excluded at the time of our patients’ assessment. Inhalation technique is periodically evaluated by nurses or doctors in each patient. Unfortunately, in Italy an individual prescription database is not available and thus we cannot assess patients’ use of medication. In two cases, the filtering process eventually identified GER and rhinosinusitis, but poor control of asthma persisted even after comorbidities were treated. In all subjects, inhaler skills, treatment adherence, and environmental exposure to indoor/outdoor allergens as well as to second- and third-hand smoke were excluded as cause of lack of control. Eventually, three out of four patients started anti-IgE treatment; asthma control was obtained and maintenance drugs were progressively reduced. In the case that refused omalizumab therapy, pulmonary function, clinical features and controller treatment including high-dose ICS were unchanged.

Previous studies have highlighted an association between increasing asthma severity in children and reduced QoL [11–13]. Uncontrolled asthma symptoms not only affect children physically, but can impair them socially, emotionally, and educationally [13]. In line with previous observations, 3 out 4 of our cases had poor QoL, assessed by a standardized questionnaire [14]. It is well known that improving QoL in difficult asthma is not an easy task, despite a variety of treatments aimed at achieving control [12], and much more remains to be done to address the problem. Nevertheless, 2 of our 3 cases showed a remarkable improvement of QoL after one year of treatment with omalizumab.

Reduction in forced expiratory volume in the first second (FEV_1_) is often used to define childhood asthma severity in treatment guidelines and clinical studies [5, 11, 15]. Nevertheless, children with severe asthma often have a normal FEV_1_ that does not improve after bronchodilators, indicating that spirometry may be a poor predictor of asthma severity in childhood [6, 16, 17]. Actually, children with a normal FEV_1_, both before and after β_2_-agonist, may show a bronchodilator response in terms of forced expiratory flow between 25% and 75% (FEF_25–75_) [18]. However, the utility of FEF_25–75_ in the assessment or treatment of severe asthma is currently unknown. Interestingly, all the reported cases showed normal or slightly reduced values of FEV_1_ but severe impairment of FEF_25–75_. Two cases showed a bronchodilator response in terms of FEV_1_ (subjects 3 and 4), while 3 patients had a significant increase of FEF_25–75_ (cases 1, 3 and 4). Unfortunately, we could not provide the results of bronchodilator response during or after the treatment with omalizumab in any case.

Available literature on the diagnostic approach to difficult asthma in children offers a number of reviews which basically summarize the steps needed to fill the gap between a generic diagnosis of “difficult asthma” and more specific labels (i.e., “severe” asthma, “difficult-to-treat” asthma, or even different diagnoses) [3, 5, 6, 8, 10, 19–21]. So far, few original articles and case reports have been published, probably due to the peculiarity of the issue, which makes retrospective discussion of cases easier than the design of a prospective clinical study [4, 22–26]. Available knowledge mainly derives from the experience of specialized centers.

**Fig. 1:**
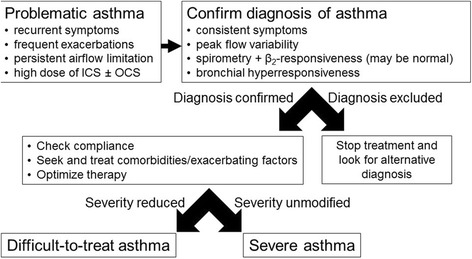
A practical algorithm for the diagnosis of difficult-to-treat and severe asthma. ICS, inhaled corticosteroids; OCS, oral corticosteroids

The evaluation of a child referred for uncontrolled asthma should start with a careful history focused on typical respiratory symptoms and on the definition of possible triggers. In the “severe asthma” process, it is crucial for clinicians to maintain a high degree of skepticism about the ultimate diagnosis, particularly in the presence of relevant discrepancies between history, physical features and lung function, as many conditions may be misdiagnosed as asthma. In order to simplify this process, herein we propose an algorithm for the diagnosis of difficult-to-treat and severe asthma (Fig. 1). Confirmation of the diagnosis through a detailed clinical and laboratory re-evaluation is important because in 12–50% of cases assumed to have severe asthma this might not be the correct diagnosis [10]. Several documents have indicated the main steps of the process that should be followed in children with uncontrolled asthma [3, 8, 10]. The translation of these procedures into real life practice may deeply change from one subject to another due to the variability of individual patients’ history and clinical features, which will often lead the diagnostic investigations towards the most likely reason for uncontrolled asthma. For children with apparently severe asthma, the first step is to confirm the diagnosis and, before proceeding to broader investigations, to verify that the poor control is not simply determined by poor adherence to treatment, inadequate inhaler skills and/or environmental exposure to triggers. A nurse-led assessment, including a home visit, despite not being applicable in all settings, may be useful for identifying potentially modifiable factors in uncontrolled pediatric asthma [27].

A number of comorbidities have been increasingly recognized as factors that may impact asthma clinical expression and control in childhood [10, 28]. Children with uncontrolled disease should be investigated for GER, rhinosinusitis, dysfunctional breathing and/or vocal cord dysfunction, obstructive sleep apnea, obesity, psychological factors, smoke exposure, hormonal influences, and ongoing drugs [3, 6, 8, 20]. Indeed, the exact role played by comorbidities in pediatric asthma control is still debated [28]. The most impressive example is GER. Several pediatric documents recommend assessing for GER because reflux may be a contributing factor to problematic or difficult asthma [7, 29]. Nevertheless, GER treatment might not be effective for severe asthma [30, 31], as confirmed by current cases 1 and 2. There is an established evidence that chronic rhinosinusitis is associated with more severe asthma in children [32–34]. Therefore, examination of upper airways and *ad hoc* treatment if rhinosinusitis is evident are recommended in children with severe asthma [3, 8, 35]. However, intranasal steroids for rhinitis resulted in a small reduction of asthma risk in school-aged children [36], and actual placebo-controlled studies on the effect of treatment of rhinosinusitis on asthma control in children are lacking [10, 37].

Dysfunctional breathing, including hyperventilation and vocal cord dysfunction, is associated with poorer asthma control in children [8, 10, 38, 39]. Unfortunately, there is scarce literature on the effect of its treatment on the control of severe asthma in children [40]. SDB ranging from primary snoring to obstructive sleep apnea syndrome is very common in children [41], and an increased prevalence of SDB together with increasing asthma severity has been reported [42]. Interestingly, GER may also be worsened by recurrent episodes of upper airway obstruction associated with SDB, and this may further trigger bronchial obstruction. Asthma guidelines recommend the assessment of SDB through nocturnal polysomnography in poorly controlled asthmatics, particularly if they are also obese [5]. There are no studies examining whether pediatric asthma improves after SDB has been treated, for example, with nasal steroids, adenotonsillectomy, continuous positive airway pressure or weight reduction if the child is also obese [43]. The parallel increase in obesity and asthma suggests that the two conditions are linked and that they can aggravate each other [44, 45], even though the exact mechanisms that underlie this association remain unclear [46]. Indeed, other coexisting comorbidities such as SDB or GER may play a confounding role in the development of the interactions between obesity and the airways [47, 48]. Obesity is associated with increased markers of inflammation in serum and adipose tissue and yet decreased airway inflammation in obese people with asthma [49]. Several interventions, including behavioral and weight reduction programs or bariatric surgery, may result in improved asthma control, quality of life and lung function in adult obese asthmatics [50]. Although reports of adolescent bariatric surgery demonstrate a significant body weight decrease, this approach is not widely available and there are no published reports on its effect on pediatric severe asthma control [51]. Finally, although it is still unclear whether food allergy is causative or shares a common pathway with difficult asthma, it might explain the loss of asthma control at least in some children and thus be considered as a comorbid condition [10, 16, 52].

In conclusion, establishing the impact of comorbidities on asthma control may be cumbersome, and an ex-juvantibus treatment is sometimes necessary to assess their role. Comorbid conditions can also worsen each other, and symptoms arising from some of them may mimic asthma [6]. Although the ability to improve pediatric severe asthma by treating comorbidities remains unconfirmed, they should be treated appropriately [9].

**Fig. 2:**
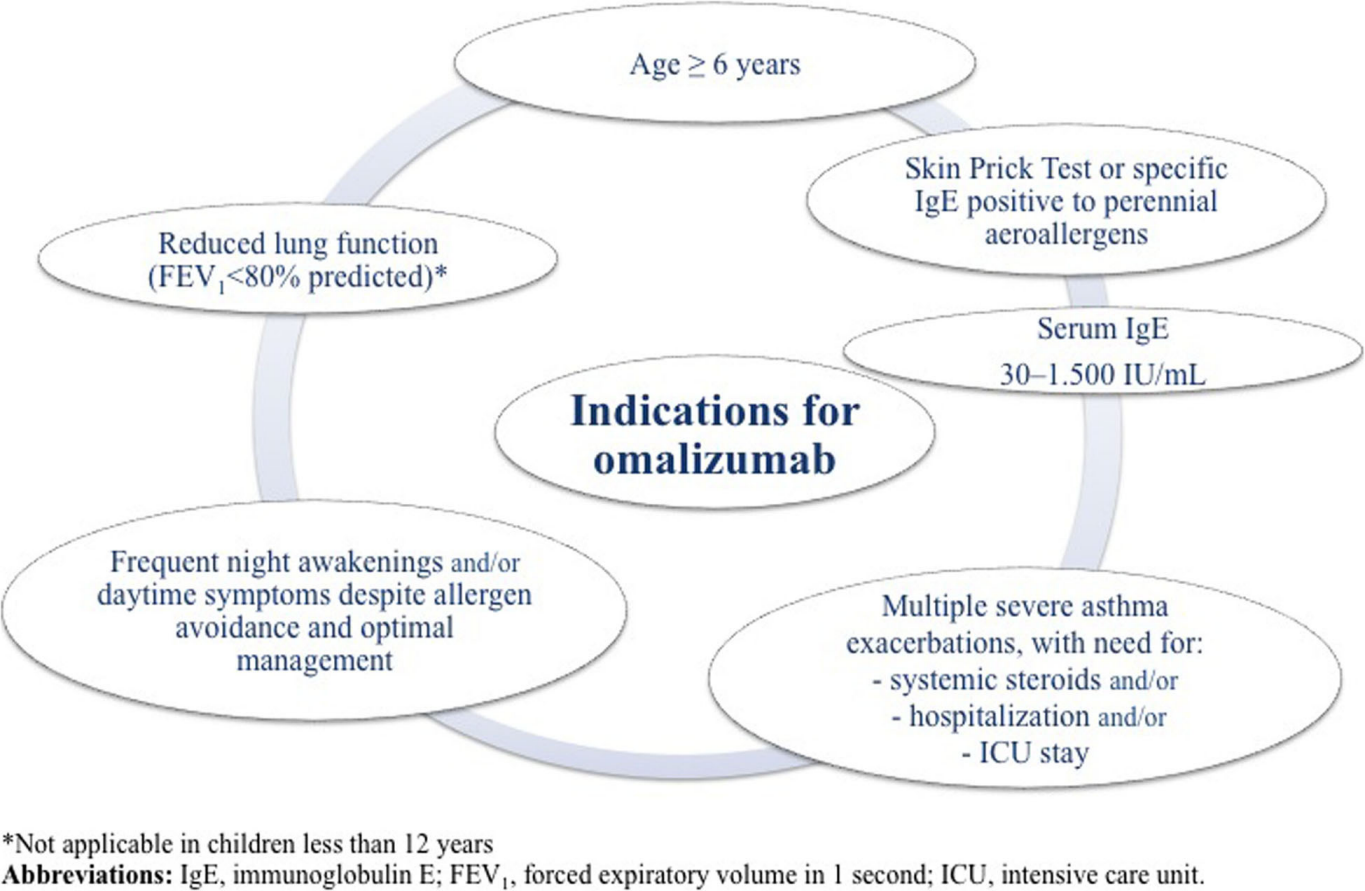
Indications for omalizumab in children and adolescents with severe asthma

The vast majority of asthmatic children exhibit a mild or at most a moderate disease that can be fully controlled with low-to-medium dose ICS associated or not with other controllers [5, 6]. However, a subset of asthmatics remains difficult-to-treat [5, 6]. With the advent of biologics, these severe steroid-dependent asthmatics have alternative options for treatment, as steroid-related adverse events are common in severe asthma [53]. Omalizumab, an anti-IgE monoclonal antibody, is the only biologic therapy recommended in children with moderate-to-severe asthma by the recent guidelines [5, 6]. In Italy, this treatment is fully covered by the National Health System. Therefore, there is no influence by any funding on treatment decisions. It was approved by the US (Food and Drug Administration) in 2003 and by the European Union (European Medicines Agency) in 2005 as an add-on treatment for patients aged > 12 years with severe persistent allergic asthma and who have a positive skin test or in-vitro reactivity to a perennial aeroallergen, FEV_1_ < 80% predicted, frequent daytime symptoms or nighttime awakenings, and multiple documented severe asthma exacerbations despite daily ICS *plus* a LABA [54, 55]. In 2009, it also received approval in Europe for treating patients aged 6–12 years. Figure 2 illustrates current indications for treatment with omalizumab in children and adolescents with severe asthma.

IgE antibodies, Th_2_-derived cytokines and eosinophils play a major role in the development of chronic airway inflammation in asthmatic subjects [56]. Once released from plasma cells, IgE binds principally to the high-affinity IgE receptor (FcεRI) on mast cells, triggering different effector responses, including the release of mediators leading to allergic inflammatory reactions [56]. The activation of the allergic cascade by IgE, under constant allergen stimulation, leads to the establishment of chronic allergic inflammation in the airways of asthmatic patients, with IgE being a key element of the vicious circle that maintains it. Cytokines produced during the late phase and subsequent chronic inflammation stage have been directly associated with the induction of airway remodelling, indirectly implicating IgE in the process [56]. At present, omalizumab is the only commercially available recombinant humanized anti-IgE monoclonal antibody that specifically binds serum free IgE at its CH_3_ domain, in the proximity of the binding site for FcεRI, thus preventing IgE from interacting with its receptor on mast cells, basophils, antigen-presenting cells and other inflammatory cells [57]. The rapid reduction of free IgE levels leads to a downregulation of the FcεRI expression on inflammatory cells and an interruption of the allergic cascade, which results in the reduction of peripheral and bronchial tissue eosinophilia and of levels of granulocyte macrophage colony stimulating factor, interleukin (IL)-2, IL-4, IL-5, and IL-13 [58]. Moreover, basophils have a relevant role in the initiation and progression of allergic inflammation, suggesting that they may represent a viable therapeutic target. Indeed, in children with severe asthma, it has been reported that omalizumab therapy is associated with a significant reduction in circulating basophil numbers, a finding that is concurrent with improved clinical outcomes [59]. This finding supports a mechanistic link between IgE levels and circulating basophil populations, and may provide new insights into one mechanism by which omalizumab improves asthma symptoms.

Several clinical controlled and real-life studies of adults with severe, inadequately controlled allergic asthma have demonstrated the efficacy and safety of omalizumab in reducing asthma-related symptoms, corticosteroid use, exacerbation rates, and healthcare resource utilization, and in improving QoL and lung function [60–63]. Fewer studies have been published in children. In two double-blind, randomized, placebo-controlled trials (RCTs) of children aged 6 to 12 years with moderate-to-severe allergic asthma, treatment with omalizumab reduced the requirement for ICS and protected against disease exacerbations, but there was little change in asthma symptom scores or spirometry [9, 64]. These findings were confirmed and extended in older children [65–67].

The results of the ICATA study, a multicenter RCT of 419 inner-city children, adolescents and young adults with persistent allergic asthma, showed that, compared to placebo, omalizumab reduces the number of days with asthma symptoms and the proportion of participants with at least one exacerbation by approximately 25% and 19%, respectively (*p* < 0.001), thus reducing the need for asthmatic symptom controllers [68]. Another multicenter RCT of inner-city children and adolescents showed that the addition of omalizumab to ongoing guidelines-based care before patients return to school reduces fall asthma exacerbations (odds ratio, 0.48), particularly in subjects with a recent exacerbation [69]. Moreover, in a real-life study of 104 children and adolescents with severe allergic refractory asthma followed over 1 year, treatment with omalizumab resulted in good asthma control in 67% of the cases (*p* < 0.001), while FEV_1_ improved by 4.9% (*p* = 0.02) and exacerbation rates and healthcare utilisation decreased approximately by 30% (*p* < 0.001) [70]. The same authors also showed that, after two years of treatment, exacerbation rate and healthcare utilisation were further decreased by 83% and 100%, respectively, while level of asthma control, steroid use and lung function remained unchanged [71].

A systematic review of pediatric RCTs pooled the data of 1381 children and adolescents with moderate-to-severe allergic asthma in order to establish the efficacy of omalizumab as an add-on therapy [72]. During the stable-steroid phase, omalizumab decreased the number of patients with at least one exacerbation (risk ratio, 0.69; *p* < 0.001), the mean number of asthma exacerbations per patient (risk ratio, 0.35; *p* < 0.001), and the asthma symptom score (mean difference, 0.12; *p* = 0.005) when compared to placebo. During the steroid reduction phase, omalizumab further reduced the number of patients with at least one exacerbation (risk ratio, 0.48; *p* < 0.001) and the mean number of asthma exacerbations per patient (mean difference, 0.12; *p* < 0.05).

Given the cost of omalizumab, many authors have argued for the importance of identifying specific asthma populations who will have significant benefit from it [68, 73, 74]. In the ICATA study, baseline predictors of good response to treatment were sensitization and exposure to cockroach allergen, sensitization to house dust mite allergens, a serum IgE level of more than 100 IU per milliliter, a BMI of 25 or more, and a history of at least one unscheduled medical visit in the previous year [68].

Several studies have assessed the long-term safety of omalizumab in children and adults. A pooled analysis of 67 RCTs conducted over 2 decades on 4254 children and adults treated with omalizumab showed no association between omalizumab treatment and risk of malignancy [75]. In an RCT evaluating 225 school-aged children, omalizumab was well tolerated, there were no serious adverse events, and the frequency and types of all adverse events were similar to the placebo group [9]. These results have been further confirmed by a recent systematic review of RCTs that concluded that treatment with omalizumab does not result in increased risk of malignancy or hypersensitivity reactions [72].

While the rationale for long-term treatment with omalizumab is supported by pharmacokinetic-pharmacodynamic models [76], the duration of treatment is still under discussion. Results from published studies suggest that omalizumab should be continued for > 1 year [77, 78]. In a retrospective study of adults and children with uncontrolled severe asthma treated with omalizumab, the response to treatment was ‘excellent’ in 52.5% of patients, particularly in the subgroup of children aged 6 to 11 years [77]. After the discontinuation of treatment, loss of asthma control was documented in 69.2% of the patients who had received omalizumab for < 1 year, 59.1% of the subjects treated for 1–2 years, and 46.1% of the cases treated for > 2 years. Time to loss of control was shorter in younger children and longer in patients with an ‘excellent’ response compared with patients with a ‘good’ response. No early loss of control (within 6 months) was observed among patients with > 3.5 years of continuous treatment with omalizumab. Finally, 20% of patients in whom omalizumab was re-prescribed because of loss of control did not respond to the treatment anymore [77]. Despite these encouraging findings, the impact of omalizumab on the natural history of severe asthma in children deserves to be further investigated by long-term studies that will also define the criteria and timing for discontinuing the treatment.

It is well known that asthma pharmacotherapy is effective in controlling symptoms and bronchial inflammation, but cannot affect the underlying immune response, thus leading to the possibility of symptom reappearance after its discontinuation [79]. In this scenario, allergen-specific immunotherapy (AIT) has been proposed as the only therapeutic method that can modulate the underlying immune pathophysiology in allergic asthma [80].

AIT is currently indicated in children and adults with mild-moderate allergic asthma that is completely or partially controlled by pharmacotherapy and with the evidence of a clear relationship between symptoms and exposure to a specific allergen [81–84]. However, according to recent guidelines, the efficacy of AIT in asthmatic subjects is limited, and its potential benefits must be weighed against the risk of side effects and the inconvenience and costs of the prolonged therapy [5]. Moreover, severe or uncontrolled asthma (regardless of its severity) is a major independent risk factor for non-fatal or even fatal adverse reactions, thus representing a contraindication for AIT [85–87]. Finally, children with severe asthma are often sensitized to multiple allergens, thus making AIT prescription even more complicated [88].

In subjects with uncontrolled and/or severe allergic asthma, a combination of omalizumab and AIT has been proposed [88]. Surprisingly, only a few studies have addressed this issue [89–92]. However, pre-treatment with omalizumab seems to improve the efficacy and tolerability of subcutaneous AIT in children and adults with severe allergic asthma both during omalizumab treatment and after its discontinuation [89, 91, 92]. Omalizumab has also been successfully used as a supplementary treatment to AIT in order to improve asthma control in children ≥6 years with severe persistent allergic asthma [90]. Given the scarcity of studies on AIT *plus* omalizumab in children with severe allergic asthma, further research is warranted to assess risks and benefits of the combined treatment.

Children with severe asthma require a detailed and individualized approach including re-assessment for differential diagnoses, comorbidities and contributory factors, environmental triggers, lung function and inflammation, adherence and response to therapy, and QoL. Treatment of pediatric severe asthma still relies on the maximal optimal use of corticosteroids, bronchodilators and other controllers recommended for moderate-to-severe disease. However, the management of asthma is becoming much more patient-specific, as more and more is learned about the biology behind the development and progression of asthma.

In the current paper, we described the characteristics of four children with severe asthma in whom omalizumab was prescribed. A review of the relevant literature on the topic was also performed. Finally, we provided an algorithm for the diagnosis of difficult-to-treat and severe asthma in children and adolescents, based on the evidence from the literature review. As all algorithms, it is not meant to replace clinical judgment, but it should drive physicians to adopt a systematic approach towards difficult and severe asthma and provide a useful guide to the clinician.

The addition of omalizumab, the first targeted biological treatment approved for asthma, has led to renewed optimism of outcome improvements in patients with allergic severe asthma. As severe asthma is a heterogeneous condition consisting of different phenotypes, the future of asthma management will likely involve phenotypic and potentially even genotypic characterization in selected cases in order to determine appropriate therapy and thus to provide the highest possible benefit, especially if specific responder phenotypes can be identified and selected for this highly specific treatment.

## Abbreviations

anti-IgE: Anti-immunoglobulin E
BMI: Body mass index
FcεRI: IgE receptor
FEF_25–75_: Forced expiratory flow between 25% and 75%
FEV_1_: Forced expiratory volume in the first second
GER: Gastroesophageal reflux
ICS: Inhaled corticosteroids
ICU: Intensive care unit
IL: Interleukin
LABA: Long-acting β_2_-agonist
LTRA: Oral leukotriene receptor antagonist
QoL: Quality of life
RCTs: Randomized controlled trials
SABA: Short-acting β_2_-agonists
SDB: Sleep-disordered breathing
